# Mung Bean Protein Hydrolysates Protect Mouse Liver Cell Line Nctc-1469 Cell from Hydrogen Peroxide-Induced Cell Injury

**DOI:** 10.3390/foods9010014

**Published:** 2019-12-23

**Authors:** Jianhua Xie, Hedan Ye, Mengxia Du, Qiang Yu, Yi Chen, Mingyue Shen

**Affiliations:** State Key Laboratory of Food Science and Technology, Nanchang University, Nanchang 330047, China; jhxie@ncu.edu.cn (J.X.); yz121786@163.com (H.Y.); GXMZHISHUI@163.com (M.D.); yuqiang8612@163.com (Q.Y.); chenyi15@ncu.edu.cn (Y.C.)

**Keywords:** mung bean, protein hydrolysates, antioxidant activity, ROS, NCTC-1469 cells

## Abstract

Mung bean is nutritious and rich in protein (19.5%–33.1%). However, there are few studies on mung bean protein active peptides so the mung bean protein hydrolysates (MBPHs) were investigated for evaluating their ability to clear intracellular reactive oxygen species (ROS) and regulating the ability of antioxidant enzymes on NCTC-1469 cells. Results showed that MBPHs, MBPHs-I (molecular weight < 3 kDa), MBPHs-II (molecular weight between 3 and 10 kDa), and MBPHs-III (molecular weight > 10 kDa) could all improve the survival rate of cells compared with the model group. MBPHs, MBPHs-I, and MBPHs-II could significantly decrease the content of lactate dehydrogenase (LDH) and reduce the generation of malonaldehyde (MDA) at a concentration of 0.4 mg/mL. Regarding the intracellular ROS, the result showed that MBPHs-I significantly reduced the production of ROS (from 58.3% to 26.6%) and had a dose-dependent relationship. In addition, the amino acid analysis showed that MBPHs-I had a balanced amino acid composition. MBPHs-I is rich in lysine but was deficient in cereals. Therefore, the hydrophobic and aromatic amino acids in MBPHs-I were high, which could improve its antioxidant activity. According to the results, MBPHs-I was the best and most potent natural antioxidant and it can contribute to drug development and medical application.

## 1. Introduction

Reactive oxygen species (ROS), including hydroxyl radicals (OH), peroxyl radicals (ROO), superoxide radicals (O_2_), hydrogen peroxide (H_2_O_2_), and singlet oxygen [1], play a crucial role in human health and are also an indispensable part of the cell of living organism composition [2]. When ROS are at a low level, ROS can effectively play several beneficial roles in the human body, for example serving as an intracellular signaling factor. However, tissues, organs, and some of the biological macromolecules will also be damaged by oxidative stress when ROS excesses the receptivity of the human body [3]. Some serious diseases, such as diabetes [4], neurodegenerative disease [5], cancers [6], and other cardiovascular diseases [7] were related to excessive oxidative stress reaction on the human body induced by ROS.

Cells basically contain a series of complex intracellular antioxidant defense systems, which are mainly composed of two parts, enzymatic and a non-enzymatic antioxidant system. The endogenous antioxidant enzymes primarily include superoxide dismutase (SOD), glutathione peroxidase (GSH-Px), catalase (CAT), and the non-enzymatic antioxidant system contains glutathione (GSH), carotenes, ascorbic acid, and so on [8]. In normal cases, cells can prevent and relieve oxidative stress by the two anti-oxidation systems [9]. For example, SOD could remove free radicals and reduce oxidative damage [10]. Even so, complex antioxidant defense systems still do not completely block the damage caused by oxidative stress [11]. Consequently, biomacromolecules, such as DNA, protein, unsaturated fatty acids [12], membrane lipids and carbohydrates [13] will be damaged due to lots of free radical accumulation in cells.

Therefore, the development and application of antioxidants have become very important for the prevention and protection of human health. In fact, there have been a variety of synthetic antioxidants, such as butylated hydroxytoluene (BHT) and hydroxyanisole (BHA), which have been applied to reduce the damage induced by oxidative stress. Some side effects are also associated with the application of drugs, which have caused some harm to the human body [14]. Therefore, the antioxidant effects of natural substances, for example, bioactive peptides, have received extensive attention in recent years [15] because of the increasing health and healing potential [16].

Food proteins could always be hydrolyzed with various proteases to obtain protein-derived peptides, which can play physiological roles in promoting healthy development. Bioactive peptides generally contain 2–20 amino acid residues and have a variety of biological activities, such as anti-oxidation [17], immune-protection [18], and ACE-inhibitory activity [13,19]. Furthermore, the preparation of antioxidant peptides has been reported from various food proteins, such as soybean [20], corn [21], barley [22], wheat [23], and chickpea [24], and their antioxidant activity has also been demonstrated.

Mung bean has been widely planted in Asia and is famous for its detoxification properties. They are rich in nutrients, composed of about 19.9%–33.1% protein of total dry weight, and have been used in many products, such as pastries and drinks. However, the main application is the starch, and the mung bean protein (MBP) is not well developed and used [25,26]. In fact, protein hydrolysates obtained from mung beans have many physiological activities, such as ACE-inhibitory activity [26,27], antioxidant activities [28], and antitumor [29]. Protein hydrolysates deserve more attention and exploration in recent years.

In our previous work, we found that the alkaline-protein hydrolysates of mung bean showed good scavenging activity for free radicals in vitro, such as 1, 1-diphenyl-2-picrylhydrazyl (DPPH), hydroxyl radical, and superoxide radical scavenging activities, reducing capacity and Fe^2+^ chelating activities [28]. This study will further explore the anti-oxidation effects of MBPHs in the cell. Normal living cells (NCTC-1469) were regarded as an experimental cell model to evaluate intracellular ROS scavenging activities of MBPHs. Meanwhile, the cellular antioxidant enzymes and the content of the total GSH were investigated.

## 2. Materials and Methods

### 2.1. Materials and Reagents

Mung beans (*Vigna radiata* L.) were purchased from the Grain and Oil Processing Company (Jilin, China). The mung bean protein (MBP) was extracted with the method of alkaline extraction and acid precipitation as previously described [30].

The NCTC-1469 adherent cell line derived from normal mouse liver was purchased from the American Type Culture Collection. Dulbecco’s modified Eagle’s medium (DMEM) was purchased from Gibco (New York, NY, USA). Fetal bovine serum (FBS) and 0.25% trypsin were purchased from the Solarbio (Beijing, China). Hydrogen peroxide (H_2_O_2_) was purchased from Alfa Aesar (Ward Hilll, MA, USA). Bicinchoninic acid (BCA), glutathione (GSH), superoxide dismutase (SOD), and malondialdehyde (MDA) assay kits were obtained from Beyotime Institute of Biotechnology (Shanghai, China). ROS and lactate dehydrogenase (LDH) assay kits were purchased from Hefei Bomei Biotechnology CO. (Heifei, China). 2′,7′-dichlorofluorescein diacetate (DCFH-DA), 2,2′-azobis (2-amidinopropane) dihydrochloride (ABAP) and phosphate-buffered saline (PBS) (0.144 M NaCl, 5 mM KCl, 8.5 mM Na_2_HPO_4_, 1.4 mM NaH_2_PO_4_, pH 7.4) were provided by Sigma-Aldrich (St. Louis, MO, USA). The water was purified by the Milli-Q water purification system (Millipore, Bedford, MA, USA). All other chemicals and solvents were of analytical reagent grade.

### 2.2. Preparation of Protein Hydrolysates and Ultrafiltered Fractions

Mung bean protein hydrolysates (MBPHs) were prepared according to the previously published method [28]. In brief, the mixture was hydrolyzed by alcalase at an enzyme/substrate ratio of 3/100 (*v*/*w*) for 2 h, then was heated at 95 °C for 10 min for enzyme deactivation. After cooling, the hydrolysate was centrifuged at 5000× *g* (4 °C) for 10 min. The supernatant was adjusted to pH 7.0. Then the supernatant was desalinated with a dialysis bag (100–500 Da) for 48 h. The dialysate was lyophilized and stored for the next studies.

The MBPHs were separated using ultrafiltration with molecular weight cut-off (MWCO) membranes of 10 kDa and 3 kDa. Three fractions with molecular weights of <3 kDa (MBPHs-I), 3–10 kDa (MBPHs-II), and >10 kDa (MBPHs-III) were collected, then they were lyophilized and stored at −80 °C.

### 2.3. Cell Culture

NCTC-1469 cells were cultured in DMEM supplemented with 10% fetal bovine serum (FBS), 100 U/mL penicillin, and 100 U/mL streptomycin. The cells were incubated at 37 °C in a humidified atmosphere of 5% CO_2_ to a confluence of 70%–90% and afterward split to a lower density.

### 2.4. Influence of Cell Oxidative Damage Induced by H_2_O_2_

The cell viability was determined using a CCK-8 assay [31]. NCTC-1469 cells in logarithmic growth were seeded into 96-well plates with density of 1 × 10^5^ cells/mL under 5% CO_2_ at 37 °C for 12 h. Then the medium was removed and the cells were treated with a new 200 μL cell medium containing different concentration of H_2_O_2_ (100–1000 μM) and the H_2_O_2_ treatment lasted for 0, 2, 4, 6, 8, and 10 h. Afterward, the medium was removed, the 96-well plate was washed twice with fresh cell medium, and 10 μL of CCK-8 reagent was added. After 1 h, the absorbance was measured under 570 nm. Then, the appropriate H_2_O_2_ concentration and time in the injury model were screened.

### 2.5. Assessment of Cytotoxicity of Protein Hydrolysates Using CCK-8 Assay

NCTC-1469 cells in logarithmic growth were seeded in a 96-well plate at a concentration of 1 × 10^5^ cells/mL. After 12 h of cultivation, the cell medium was removed. The cells were treated with various concentrations of the MBPHs, MBPHs-I, MBPHs-II and MBPHs-III (0.1, 0.2, 0.4, 0.8, 1.0, 2.0, 3.0, 4.0, 5.0 mg/mL). Then the medium was added to 200 μL. After 12 h, the medium was removed and the 96-well plate with the fresh medium was washed twice. Finally, the cell was adding 100 μL of new cell medium and 10 μL of CCK-8 solution and cultured for 1 h, the absorbance was measured at 570 nm.

### 2.6. Protection Effect of MBPHs and Three Fractions on Oxidation-Induced Cell Damage

The protective effect of MBPHs, MBPHs-I, MBPHs-II, and MBPHs-III on the injured NCTC-1469 cells by H_2_O_2_ were performed. Cells were seeded in a 96-well plate at a concentration of 1 × 10^5^ cells/mL. After cultivation for 12 h, the cell medium was removed. Then the cells were exposed to MBPHs, MBPHs-I, MBPHs-II, and MBPHs-III with different concentrations (0.1, 0.2, 0.4 mg/mL), respectively. After 12 h, the medium was removed totally, and a new medium containing H_2_O_2_ (500 μΜ) was added for another 4 h. Following, the medium was removed and the 96-well plate with the fresh medium was washed twice. The model group was treated with H_2_O_2_.

### 2.7. Determination of MDA, GSH, SOD, and LDH

NCTC-1469 cells (1 × 10^6^ cell/well, 2 mL/well) were cultivated in 6-well plates and incubated with MBPHs-I and MBPHs-II (0.1, 0.2, 0.4 mg/mL) under the conditions mentioned above. The level of SOD, MDA, and LDH in cells were determined by the kits. The content of GSH was evaluated as described [32].

### 2.8. Determination of Intracellular ROS

The secretion of ROS by NCTC-1469 cells was tested using 2, 7-dichlorofluorescein diacetate (DCFH-DA) fluorescence assay [31,33]. NCTC-1469 cells (1 × 10^5^ cell/well, 200 μL/well) were seeded in 96-well plate and incubated with MBPHs-I (0.1, 0.2, 0.4mg/mL) for 24 h and then transferred to a EP tube (microcentrifuge tube for separation of trace reagents) of 1.5 mL, which was centrifuged using at 1500× *g* for 5 min. The supernatant was removed and the cells were washed using PBS for twice, then 100 μL of PBS was added aimed to resuspend cells. Aliquot of 10 μM DCFH-DA fluorescent probe solution was added in three groups and the resulting mixture was incubated for 0.5 h at 37 °C. After washing twice with PBS to remove the extracellular DCFH-DA, the cells were resuspended into a single cell suspension. Finally, the intracellular ROS level was determined with a BD FASCalibur flow cytometer (Franklin Lakes, NJ, USA).

### 2.9. Amino Acid Analysis

Amino acid compositions of two components after ultrafiltration were analyzed using a modified method described [34]. Briefly, the total amino acid compositions of the samples were determined with an Automatic Amino Acid Analyzer (L-8900, Hitachi, Tokyo, Japan) after hydrolysis with 6 M HCl at 110 °C for 22 h.

### 2.10. Statistical Analysis

Data were all performed in triplicates and presented as mean ± standard deviation (SD). One-way analysis of variance (ANOVA) followed by multiple comparisons with least significant difference (LSD) tests were performed. The significant difference was analyzed among samples by SPSS (Version 19.0, IBM Company, USA) at *p* < 0.05.

## 3. Results and Discussion

### 3.1. Cell Model Induced by H_2_O_2_

**Figure 1:**
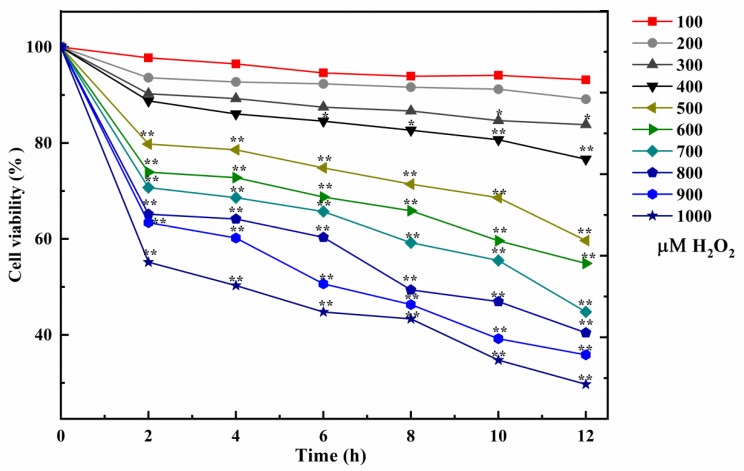
Effect of H_2_O_2_ on the survival rate of NCTC-1469 cells (*n* = 6). Results are expressed as means ± SD (*n* = 6), * *p* < 0.05, ** *p* < 0.01.

H_2_O_2_ is generated in the mitochondrial respiratory chain and produced by the disproportionation effect of O^2-^ [35,36,37]. The hydroxyl radical could be quickly offered by H_2_O_2_ in Fenton chemical changes. The hydroxyl radical is one of the most common free radicals, which will induce lipid oxidation, bio-macromolecule damage, lead to mitochondrial dysfunction and calcium imbalance, and cause cell apoptosis. Therefore, it is the key to select the appropriate concentration and acting time of H_2_O_2_. The activity of the NCTC-1469 cells is evaluated using CCK-8 analysis, and the results showed the number of living cells gradually decreased when the concentration and time of H_2_O_2_ increased in Figure 1. When the H_2_O_2_ concentration was from 500 to 1000 μΜ, cultivated with all times, the percent of living cells significantly decreased (** *p* < 0.01). It is worth noting that when the concentration was 500 μΜ and the action time was 4 h, 25.0% of the cell viability was inhibited, which was expressed as a decrease in cell activity with a significant difference (** *p* < 0.01). Therefore, 500 μΜ was regarded as the optimal concentration of our model group and 4 h as the optimal induction time.

### 3.2. Cytotoxicity of Mbphs and the Ultrafiltration Fractions

**Figure 2:**
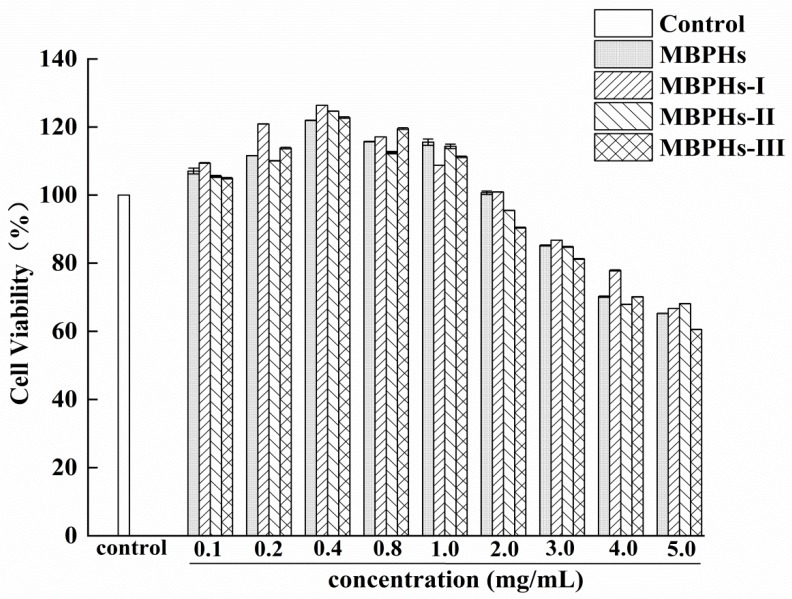
Evaluation of toxicity of mung bean protein hydrolysates (MBPHs), MBPHs-I, MBPHs-II, and MBPHs-III on the injured NCTC-1469 cells. Results are expressed as means ± SD (*n* = 6).

MBPHs, MBPHs-I, MBPHs-II, and MBPHs-III were used to treat injured NCTC-1469 cells for evaluating their cell toxicities, respectively. The result was displayed in Figure 2. Compared with the normal group, MBPHs, MBPHS-I, MBPHs-II, and MBPHs-III promoted the cell viability at the concentrations (from 0.1 to 2.0 mg/mL). At the concentration of 0.4 mg/mL, the cell survival rate was 120%. The effect of MBPHs-I was better than other groups and there was a significant difference compared with the normal group. It showed an inhibitory effect when the concentration continued to increase. Therefore, the concentrations of mung bean protein hydrolysates (0.1–2.0 mg/mL) were determined to be used in the further antioxidant test.

### 3.3. Protective Effects of MBPHs and Ultrafiltration Fractions

**Figure 3:**
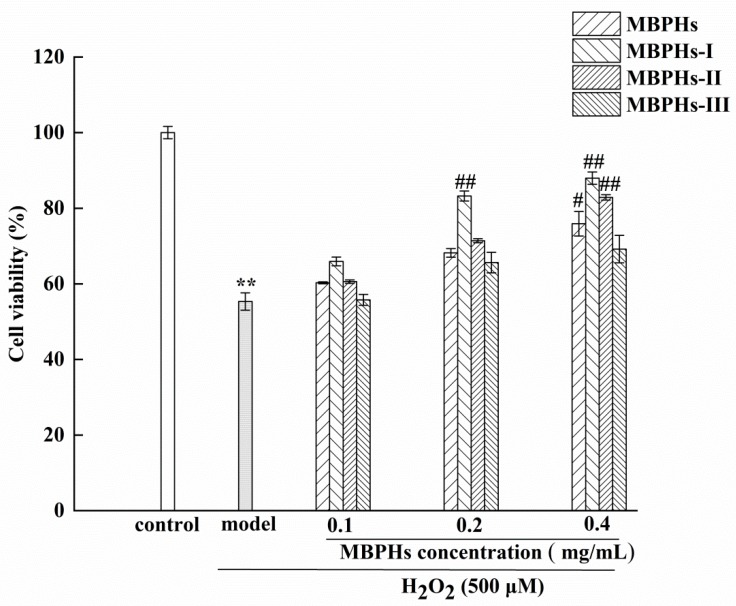
Cytoprotective effects of MBPHs, MBPHs-I, MBPHs-II, and MBPHs-II on H_2_O_2_-induced oxidized NCTC-1469 cells. Results are expressed as means ± SD (*n* = 6), ** *p* < 0.01 vs. control group, ^#^ *p* < 0.05 vs. model group, ^##^ *p* < 0.01 vs. model group.

The protective effect on the viability of H_2_O_2_-induced NCTC-1469 cells was determined using the CCK-8 solution. As shown in Figure 3, the viability of the cells decreased significantly after culturing with H_2_O_2_ for 4 h compared with the control group (*p* < 0.01). After adding MBPHs, MBPHs-I, MBPHs-II, and MBPHs-III, the viability of the cells increased to varying degrees. When the dose was 0.4 mg/mL, the cell survival rates of the four components were 78%, 86%, 83%, 74%, respectively. The protective effect of MBPHs-I in the cells induced by H_2_O_2_ was higher than that of other components, and the intensity was MBPHs-I > MBPHs-II > MBPHs > MBPHs-III. In addition, there was a dose-dependent relationship between cell viability and the concentrations of every protein hydrolysate. Therefore, we further studied the anti-oxidation effects of MBPHs-I and MBPHs-II.

### 3.4. Changes of MDA, GSH, SOD, and LDH on Injured NCTC-1469 Cells

Oxidative cellular stress is induced by ROS which usually can result in lipid peroxidation, even cell death [38,39], which is generally related to the cleavage of polyunsaturated fatty acids [40].

**Figure 4:**
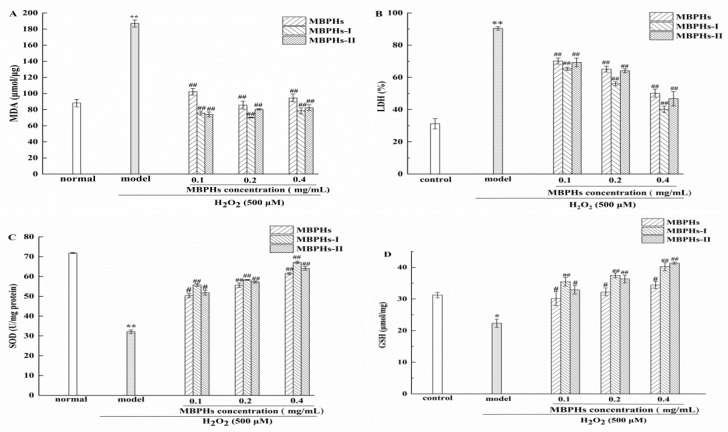
Protective effects of MBPHs, MBPHs-I, and MBPHs-II from mung bean on the H_2_O_2_-induced NCTC-1469 cells. (**A**) Effect on the malonaldehyde (MDA) level; (**B**) effect on the activity of lactate dehydrogenase (LDH); (**C**) effect on the activity of superoxide dismutase (SOD); (**D**) effect on the glutathione (GSH) content. Results shown are expressed as means ± SD (*n* = 6), * *p* < 0.05, ** *p* < 0.01 vs. control group, ^#^ *p* < 0.05 vs. model group, ^##^ *p* < 0.01 vs. model group.

MDA is one of the most important products of membrane lipid peroxide and its production can also aggravate the damage of the cell membrane. The content of MDA can indirectly reflect the damage degree of the membrane system [41]. So, the content of MDA was determined and presented in Figure 4A. We could see that the MDA levels of the model group extremely significantly increased compared with the normal group (*p* < 0.01) which means the cell membrane has been severely damaged because of membrane lipid peroxide occurring. Among samples, MBPHs-I exhibited the best inhibitory effect, particularly when the concentration was 0.2 mg/mL. However, the MDA contents of sample groups had no significant difference at 0.2 and 0.4 mg/mL. When the organs are damaged, LDH is released into the blood, so LDH release can reflect the integrity of the cell membrane [42]. The ability of MBPHs to protect the cell membrane can be evaluated via determining the level of LDH. As shown in Figure 4B, the LDH levels of the model group displayed significantly augment compared with the normal group (*p* < 0.01), which indicated that H_2_O_2_ caused damage to NCTC-1469 cells. When the cells were cultured with MBPHs, MBPHs-I, and MBPHs-II, the generation of LDH got significant inhibition compared with the model group (*p* < 0.01). Among samples, MBPHs-I exhibited the best inhibitory effect. Particularly when the concentration increased 0.4 mg/mL, the production of LDH was statistically downregulated close to the normal group (Figure 4B). The number of LDH was decreased to 40.21% when the sample was 0.4 mg/mL. The result indicated that MBPHs, MBPHs-I, and MBPHs-II all had potential effects to attenuate ROS-mediated membrane damage to achieve a better protective effect on NCTC-1469 cells injured model, and MBPHs exhibited the best effect of reducing MDA levels.

In addition, another key action of cytoprotective candidates is an enhancement of the intrinsic antioxidant defense system. SOD is an important member of the antioxidant enzyme system in the biological system. [43]. The superoxide anion combined with ·OH will cause DNA damage and destroy the function of the human body. However, SOD can effectively eliminate O_2_, which protect the body from the influence of superoxide anion. [44]. As shown in Figure 4C, the level of SOD significantly decreased (*p* < 0.01) after the incubation with H_2_O_2_ (500 μΜ) for 4 h. The SOD content of every concentration of MBPHs, MBPHs-I, and MBPHs-II was significantly improved compared with the model group (*p* < 0.01). The sample groups all had a dose-dependent concentration relationship. GSH can bind to free radicals to alleviate the oxidative damage of free radicals. Moreover, GSH (especially in live-cells) can participate in biotransformation, thus converting harmful toxins into harmless substances in the body and excreting them out of the body. [45,46,47]. As shown Figure 4D, after the incubation with H_2_O_2_ (500 μΜ) for 4 h, GSH significantly declined (from 31.23 ± 0.89 μmol/mg to 22.32 ± 1.32 μmol/mg) compared with the normal group (*p* < 0.05), which suggested that the NCTC-1469 cells were injured seriously. However, the treatment of samples significantly improved the levels of GSH and had a significant enhancement compared with the model group. In particular, the contents of GSH were the highest at the concentration of 0.4 mg/mL. The results suggested that mung bean protein hydrolysates could enhance antioxidant enzyme activity to mitigate the damage caused by oxidative stress.

Similar results have been shown in previous studies, such as SPH-I (Mw < 3 kDa) from the alcalase-hydrolyzed soybean hydrolysate (SPH) which decreased the death rate of injured Caco-2 cells induced by oxidative stress, increased CAT activity, and decreased MDA content [48]. Feng et al. [49] reported that two peptides (TY and SGGY) from defatted walnut meal protein hydrolysates (DEMPH) had the protective effect against oxidative damage to SH-SY5Y cells, therefore SGGY could significantly increase cell viability, and improve GSH content at a concentration of 1.0 mg/mL (*p* < 0.01). Therefore, all the results suggested that mung bean protein hydrolysates exerted a protective effect on the injured NCTC-1469 cells through the inhibition of lipid peroxides and increasing antioxidant enzyme activity.

### 3.5. Effect of MBPHs-I on the Level of ROS on Cells

**Figure 5:**
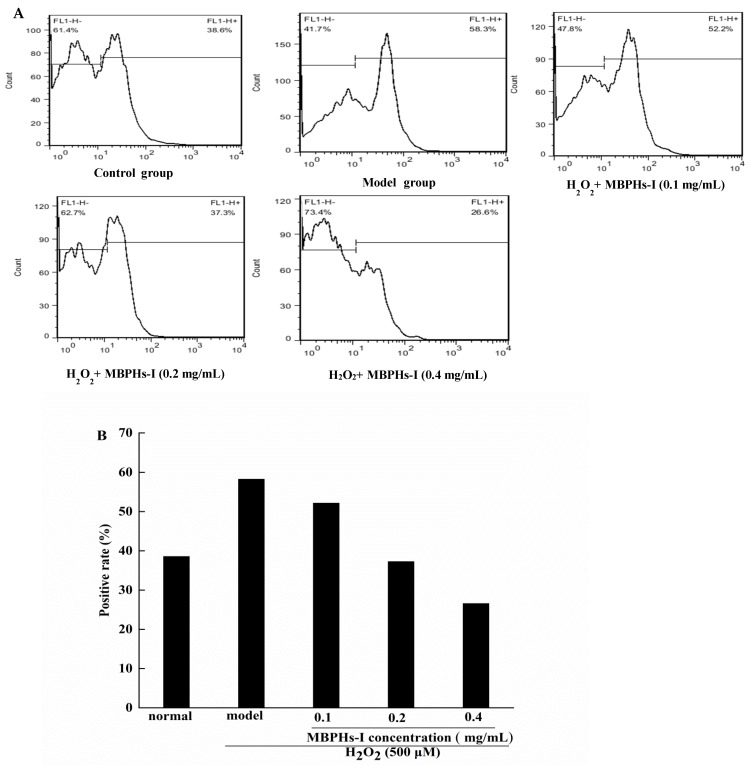
Effect of MPHS-I on reactive oxygen species (ROS) production in NCTC-1469 cells. (**A**) The flow cytometry of the effect of MBPHs-I on ROS production in NCTC-1469 cells; (**B**) a histogram of the effect of MBPHs-I on ROS generation in NCTC-1469 cells. Results shown are expressed as means ± SD (n = 6).

CFH-DA is a nonpolar dye and does not have fluorescence that can freely cross the cell membrane, but the DCFH-DA probe can be cleaved to DCFH by cellular esterases after diffusing into cells and being oxidized to DCF by peroxyl radicals produced by ABAP. DCF is based on fluorescence intensity and can be analyzed qualitatively or quantitatively. Therefore, the level of intracellular ROS can be reflected by the fluorescence intensity of DCF. The result was shown in Figure 5. Flow cytometry showed that the positive control of the model group was 58.3% and there was a much higher difference than the control group (38.6%). However, pre-treatment with MBPHs-I effectively prevented ROS generation. With the increase of concentrations, the effects of inhibition were also constantly strengthened. When the concentration of MBPHs-I was 0.1, 0.2, 0.4 mg/mL, the positive cell rates were 52.2%, 37.3%, and 26.6%. This indicted that MBPHs-I could effectively protect NCTC-1469 cells from the damage of free radicals.

### 3.6. Analysis of Amino Acid Composition of MBPHs and MBPHs-I

Mung bean is very suitable as a source of human protein and mung bean protein hydrolysate has a balanced amino acid composition. The bioactive activities of MBPHs fraction might be related to their amino acid composition. MBPHs-I had the best protective effect on cells. Therefore, the amino acid composition of MBPHs and MBPH-I needs to be determined and analyzed. In Table 1, although there are some differences in the amino acid composition between the two samples, both were rich in aspartic acid (Asp), cysteine (Cys), glutamic acid (Glu), glutamine, leucine (Leu), arginine (Arg), and lysine (Lys). There were also many hydrophobic amino acids in MBPHs and MBPHs-I, such as glycine (Gly), tyrosine (Tyr), valine (Val), methionine (Met), phenylalanine (Phe), isoleucine (Ile), leucine (Leu), and proline (Pro). The proportion of hydrophobic amino acids in MBPHs-I (38.32%) was higher than that in MBPHs (32.9%). Many studies have shown that there several hydrophobic amino acids, such as Tyr, Phe, Leu, Trp, and Pro were generally accepted as antioxidants that contribute to the scavenging of free radicals [50,51]. It has been proven that hydrophobic amino acids played an indispensable role in antioxidant activity and can improve antioxidant activity [52,53]. It is also worth noting that the content of aromatic amino acids in MBPHs-I, such as phenylalanine, tryptophan, and tyrosine, accounted for 10.57%. Amino acids with aromatic residues can donate protons to electron-deficient radicals, which may lead to higher biological activity. From another point of view, MBPHs-I had a small molecular weight that should have a lot of small molecules of polypeptides and N-terminal and C-terminal amino acid residues, which were also thought to be quite vital for antioxidant properties. In fact, it has been reported that low-molecular weight peptides could display better radical-scavenging activities than high-molecular-weight counterparts [54].

## 4. Conclusions

In the study, MBPHs were obtained by the enzymatic hydrolysis of mung bean protein and three components, MBPHs-I, MBPHs-II, and MBPHs-III, were isolated and purified by ultrafiltration membranes. Results confirmed that MBPHs, MBPHs-I, MBPHs-II, and MBPHs-III all improved cell vitality. MBPHs-I and MBPHs-II might reduce oxidative stress on NCTC-1469 cells by increasing the SOD and GSH levels and inhibiting lipid peroxidation. MBPHs-I could effectively eliminate ROS on injured NCTC-1469 cells, which might be related to high contents of hydrophobic amino acids and aromatic amino acids. All data provides that the MBPHs have an excellent protective effect against the oxidative stress by H_2_O_2_.

## Figures and Tables

**Table 1:** Amino acid composition of MBPHs and MBPHs-I.

Amino Acid	MBPHs (g/100 g)	MBPHS-I (g/100 g)
Aspartic (Asp)	9.580	11.292
Threonine (Thr)	3.022	3.319
Serine (Ser)	6.618	6.618
Glx ^a^	20.251	22.231
Alanine (Ala)	3.964	4.615
Cysteine (Cys)	10.307	10.059
Valine (Val)	4.807	5.499
Methionine (Met)	1.107	1.650
Isoleucine (Ile)	3.816	4.928
Leucine (Leu)	7.317	9.151
Tyrosine (Tyr)	3.072	4.653
Phenylalanine (Phe)	5.831	6.913
Histidine (His)	3.287	3.044
Lysine (Lys)	6.558	6.913
Arginine (Arg)	7.251	7.325
Proline (Pro)	4.047	3.790
HAA ^b^	32.904	38.317
AAA ^c^	8.903	10.566

^a^ Glutamic (Glu) and glutamine (Gln). ^b^ Hydrophobic amino acids. ^c^ Aromatic amino acids.
